# Comprehensive Mitigation of Peripheral and Central Stress Responses by Nx4: Insights From EEG and Heart Rate Variability in Post‐Stress Resting State

**DOI:** 10.1002/hup.70020

**Published:** 2025-10-07

**Authors:** Marina Krylova, Sarah Alizadeh, Hamidreza Jamalabadi, Igor Izyurov, Tara Chand, Johan van der Meer, Johannes C. Vester, Britta Naschold, Myron Schultz, Veronika Engert, Martin Walter

**Affiliations:** ^1^ Department of Psychiatry and Psychotherapy Jena University Hospital Jena Germany; ^2^ Department of Psychiatry and Psychotherapy University of Tübingen Tübingen Germany; ^3^ Department of Psychiatry and Psychotherapy Philipps‐Universität Marburg Marburg Germany; ^4^ Department of Humanistic Studies Indian Institute of Technology (BHU) Varanasi India; ^5^ Department of Radiology and Nuclear Medicine Amsterdam University Medical Center Amsterdam the Netherlands; ^6^ idv Data Analysis and Study Planning Gauting Germany; ^7^ Biologische Heilmittel Heel GmbH Baden‐Baden Germany; ^8^ Institute of Psychosocial Medicine Psychotherapy and Psychooncology Jena University Hospital Jena Germany

**Keywords:** autonomic nervous system, electroencephalography, heart rate variability, natural multicomponent multitarget medication, psychosocial stress, resting state

## Abstract

**Objective:**

Stress from daily psychosocial challenges is a significant health concern with limited pharmacological treatment options. Psychosocial stress triggers distinct responses in the autonomic and central nervous systems, measurable via heart rate variability (HRV) and electroencephalogram (EEG). This post hoc analysis of clinical trial data explores the impact of the anti‐stress medication Neurexan (Nx4) on HRV and EEG signals, and their correlation in a resting state following acute psychosocial stress induction.

**Methods:**

Data from the NEURIM trial (NCT02602275), a randomized, placebo‐controlled, double‐blind, cross‐over study, were utilized. Participants received Nx4 before exposure to ScanSTRESS, a psychosocial stress paradigm. EEG and photoplethysmogram data were collected at rest before and after stress exposure. Stress responsivity under both placebo and Nx4 conditions was evaluated through HRV and EEG signals.

**Results:**

Psychosocial stress altered HRV parameters (increased LF/HF ratio, elevated Baevsky's Stress Index, reduced RMSSD) and EEG activity (decreased aperiodic offset, increased alpha power). Nx4 significantly mitigated stress‐induced changes in LF/HF ratio, Baevsky's Stress Index, and aperiodic offset. A significant correlation was observed between Nx4 effects on HRV and EEG activity.

**Conclusion:**

Nx4 attenuates peripheral and central physiological stress responses, suggesting a comprehensive approach to mitigating stress responses in daily life.

**Abbreviations**
ANS: Autonomic nervous system
CWL: Carbon Wire Loops
ECG: Electrocardiogram
EEG: Electroencephalography
FOOF: Frequency Offset and Order Fitting
FOOOF: Fitting Oscillations and One‐Over‐F
HPA: Hypothalamic‐Pituitary‐Adrenal Axis
HRV: Heart Rate Variability
IBI: Inter‐Beat Intervals
LF/HF: Ratio Between Power in Low Frequency (LF, 0.04–0.15 Hz) and High Frequency (HF, 0.15–0.40 Hz)
PNS: Parasympathetic Nervous Systems
RM ANOVA: Repeated Measures Analysis of Variance
RMSE: Root Mean Square Error
RMSSD: Root Mean Square of Successive Differences Between Adjacent IBI Intervals
SI: Baevsky's Stress Index
SNS: Sympathetic Nervous System
TSST: Trier Social Stress Test

## 1 Introduction

### 1.1 Stress Exerts Peripheral and Central Stress Responses

Psychosocial stress activates a cascade of peripheral and central responses aimed at coping with challenging situations. While most individuals are able to manage daily stressors, the cumulative effect of repeated minor stress events may negatively impact both mental and physical health (Asselmann et al. 2017). Recognizing its wide‐reaching implications, the World Health Organization has identified stress as a major health concern (World Health Organization W.H 2001), linked to multiple leading causes of mortality (Slavich et al. 2023).

Physiological responses to stress are mediated via the autonomic nervous system (ANS), especially through sympathetic nervous system (SNS) activation, and are reflected in alterations in heart rate and heart rate variability (HRV) (Shaffer and Ginsberg 2017). At rest, parasympathetic dominance promotes lower heart rate and higher HRV; stress, by contrast, increases sympathetic tone and decreases HRV (Buske‐Kirschbaum et al. 2002; Rimmele et al. 2007; Kim et al. 2018).

While peripheral markers of stress are well studied, EEG‐based characterization of stress‐related neural activity remains comparatively underexplored (Vanhollebeke et al. 2022). Acute stress has been associated with reductions in alpha power, increases in beta activity, and enhanced frontal alpha asymmetry (Giannakakis et al. 2022; Katmah et al. 2021). However, inconsistencies across studies and analysis methods have produced mixed findings.

Recent work has highlighted the importance of accounting for broad‐band aperiodic EEG activity, which reflects non‐oscillatory neural dynamics and may shift under varying cognitive and arousal states (Donoghue et al. 2022; Waschke et al. 2021). Aperiodic activity, modeled as a 1/f distribution, can influence traditional band power estimates and may confound interpretations of oscillatory EEG changes (Donoghue et al. 2020). While the degree of this confounding remains under investigation, aperiodic features such as exponent and offset have been shown to vary systematically with arousal and stress‐related changes in neural excitation‐inhibition balance (Lendner et al. 2020), supporting their relevance for analyses of stress physiology.

EEG and HRV together offer complementary views of central and peripheral stress responses (Attar et al. 2021), with their integration improving stress state classification (Ahn et al. 2019).

### 1.2 Nx4 Modulates Stress‐Related Physiology

Neurexan (Nx4) is a multicomponent natural medication used for stress‐related symptoms such as nervous restlessness and sleep disturbances (Hajak et al. 2024). Composed of herbal extracts and a mineral salt, Nx4 has demonstrated stress‐attenuating properties in animal and human studies. In both species, it has been associated with reductions in stress hormone levels, including cortisol and adrenaline (Keller et al. 2021; Doering et al. 2016). Observational studies have supported its use in real‐world settings (Hubner et al. 2009; Waldschütz and Klein 2008).

Within the NEURIM clinical trial, Nx4 reduced susceptibility to distraction (Mayer et al. 2021), modulated emotion‐related brain connectivity (Chand et al. 2022), and attenuated amygdala and anterior cingulate cortex activation in response to psychosocial stress (Herrmann et al. 2020, 2022). Post‐stress, Nx4 improved vigilance regulation and altered stress network activation, especially in individuals with high trait anxiety (Chand et al. 2021; Nanni‐Zepeda et al. 2022).

### 1.3 Objective of the Study

We present a post hoc exploratory analysis of EEG and HRV data acquired during the NEURIM trial. The goal was to examine how experimentally induced psychosocial stress impacts resting‐state EEG and HRV characteristics in healthy male participants and whether Nx4 modulates these physiological responses. Based on previous findings, we hypothesized that Nx4 would attenuate both peripheral and central stress responses and that these effects might correlate across modalities. All analyses are exploratory in nature and intended to generate hypotheses for future confirmatory studies.

## 2 Methods

### 2.1 NEURIM Clinical Trial

This post hoc analysis is derived from an exploratory clinical trial, NEURIM (ClinicalTrials.gov identifier: NCT02602275; registered on 2015‐10‐28), with its primary endpoint previously published (Herrmann et al. 2020). NEURIM was a randomized, double‐blind, placebo‐controlled, two‐period, two‐treatment crossover trial, involving 1:1 randomization of Nx4‐Placebo and Placebo‐Nx4 treatment sequences. The study included healthy males aged 31–59 years, experiencing mild to moderate chronic stress (defined by Chronic Stress Score ≥ 9 and ≤ 36 on Trier Inventory for Chronic Stress Screening Scale and a Perceived Stress Scale score > 9). Participants received a single dose of three tablets Nx4 or placebo on two study days, with a 7–35‐day washout period. EEG, fMRI, and psychological tests were conducted on both days (Supporting Information S1). The data for this post hoc analysis of EEG and HRV data were acquired at the resting state shortly after dosing (RS1) and after psychosocial stress induction (RS2). To mitigate circadian rhythm confounding, measurements were consistently performed in the afternoon, around 4 p.m.

Psychosocial stress was induced using the ScanSTRESS task (Herrmann et al. 2020; Streit et al. 2014), an adapted version of the Montreal Imaging Stress Task, itself an fMRI‐compatible adaptation of the TSST. This task encompasses various stress dimensions, including pressure to perform, time constraints, forced failure, social‐evaluative threat, uncontrollability, and unpredictability. It involves serial subtraction and mental rotation tasks, with participants under time pressure, while two experimenters in professional attire express dissatisfaction with correctness and speed via video stream. Task speed and difficulty adapt to individual performance, preventing participants from meeting expectations. Previous publications from the NEURIM trial have explored the effects of the stress task and Nx4 on neural stress network activation and resting state vigilance state (Chand et al. 2021; Herrmann et al. 2022). This post hoc analysis study specifically focuses on stress task‐induced changes in EEG and HRV at the post‐stress resting state (RS2) compared to the pre‐stress resting state (RS1).

In the NEURIM trial, a total of 39 study participants received Nx4 and placebo and all 39 completed the study. None of the participants experienced any adverse events during the trial and vital signs were within expected ranges and showed no abnormalities. There was no indication for a safety risk after a single dose treatment with three tablets of Nx4.

The following post‐hoc analyses were exploratory in nature and were not part of the pre‐registered analysis plan. As such, no formal power calculation was conducted for these analyses. For this post hoc analysis, EEG data of sufficient quality were available for 37 participants (19 receiving Nx4 first and 18 receiving placebo first). For HRV analysis, data from 37 participants were available (20 Nx4 first and 17 placebo first). The EEG‐HRV correlation analysis included data of adequate quality for both measures from 35 participants (19 Nx4 first and 16 placebo first). The number of participants through each stage of the randomized cross‐over trial is depicted in the flow diagram (Supporting Information S1). Given the within‐subject crossover design and sample size (*n* = 35), the study was sufficiently powered to detect moderate effects (e.g., Cohen's *d* ≈ 0.48 or partial *η* ^2^ ≈ 0.10) with 80% power at *α* = 0.05. However, smaller effects may have gone undetected, and the exploratory nature of several analyses without prior registration introduces a risk of false positives. Therefore, all findings should be interpreted with caution and considered hypothesis‐generating.

### 2.2 EEG and HRV Data Acquisition

During the 12‐min resting state scans, EEG and photoplethysmogram (PPG) data were concurrently collected alongside MRI acquisition. Participants were instructed to keep their eyes closed, avoid engaging in specific tasks, and stay awake. Resting‐state EEG was recorded with eyes closed to reduce ocular and visual processing artifacts and to enhance signal stability. This approach also improves the reliability of aperiodic activity measures (Li et al. 2024). EEG data were obtained using the BrainAmp MR system (Brain Products) with a 64‐channel EasyCap and a 5000 Hz sampling rate. The AFz served as the reference, and FCz as the ground electrode, with an additional channel on the participant's back for electrocardiogram (ECG) detection. To enhance EEG recording quality during simultaneous EEG‐fMRI scans, six carbon wire loops (CWLs) were added to the EEG caps (van der Meer et al. 2016), with four placed on the outer surface at left and right frontal and posterior locations, and two attached to the cables connecting the caps to the EEG amplifier (BrainAmpMR Plus). PPG data were acquired using an MRI‐compatible plethysmograph (Siemens Medical Solutions, Erlangen, Germany) with a sampling rate of 500 Hz.

### 2.3 EEG Artifact Correction

Resting state EEG data were cleaned from gradient artifacts by motion‐informed template subtraction realized by the Bergen EEG‐fMRI toolbox (Moosmann et al. 2009). Averaging involved utilizing 25 MRI artifacts in a sliding window to generate an MRI template waveform (Allen et al. 2000), with fMRI realignment parameters as interruption points based on a 0.5 mm displacement threshold. The data then underwent bandpass filtering (0.3–200 Hz) using a Finite Impulse Response (FIR) filter (−6 dB cutoff frequency) and downsampling to 1000 Hz.

Subsequently, the CWL toolbox was applied to eliminate ballistocardiograph, helium pump, and head movement artifacts (van der Meer et al. 2016). This toolbox regressed out EEG artifacts correlated with the movements recorded by each of the six CWLs using Hann tapers with overlapping windows of 6s lengths and a delay embedding of 21 ms. The data were segmented into 2s epochs, with the removal of epochs containing muscle artifacts (outliers in spectral power between 110 and 140 Hz). Channels with over 50% artifact‐containing epochs were interpolated via EEGLAB routines (Delorme and Makeig 2004).

Independent component analysis decomposition addressed components related to eye movements, heartbeat, continuous muscle activity, and residual MRI artifacts. The final step involved re‐referencing the data to the average reference for further analysis.

Technical problems during data acquisition led to the exclusion of two participants, resulting in 37 subjects for EEG analysis.

### 2.4 EEG Spectra Parametrization

The power spectrum of artifact‐free resting state EEG data was computed using a multi‐tapered Fast Fourier Transformation, employing a 2‐s Hanning window with 50% overlap, resulting in a frequency resolution of 0.5 Hz. We confined the analysis to the 1–30 Hz frequency range due to higher frequencies being more susceptible to residual MRI artifacts (refer to Supporting Information S1). Total EEG power was decomposed into aperiodic and oscillatory components using the Fitting Oscillations and One‐Over‐F (FOOOF) algorithm (Donoghue, Haller, et al. 2020) integrated into the Fieldtrip toolbox (Oostenveld et al. 2011).

In brief, FOOOF utilizes an iterative process to quantify the exponent (slope) and offset (broadband shift) of the non‐oscillatory 1/f‐like aperiodic activity. It identifies neural oscillations as Gaussian peaks above the aperiodic component, and their combination yields a periodic activity model. This approach not only separates aperiodic and oscillatory activity but also characterizes participant‐specific neural oscillations by their center frequencies, powers, and bandwidths. Given the substantial variability of neural oscillations between and within individuals (Donoghue and Watrous 2023), this method offers a more accurate assessment of periodic activity compared to standard frequency band power analysis (Donoghue, Haller, et al. 2020). Spectral parameterization covered the entire 1–30 Hz spectrum, with peak width limited between 1 and 8 Hz, minimum peak height of 0, a peak threshold of 1.5, a maximum of six peaks, and aperiodic mode set to “fixed” (no knee). The goodness of model fit was evaluated using *R* ^2^ values (group average mean ± SD: 0.977 ± 0.021—indicating good fits) and frequency‐wise absolute error (see Supporting Information S1). For statistical analysis, channel‐wise estimates of aperiodic offset and exponent, as well as modeled oscillatory power spectra, were utilized.

To accommodate variations in the presence and peak frequencies of neural oscillations (Donoghue et al. 2022), mean oscillatory alpha and beta power were additionally extracted using a ±2 Hz interval around individual peak frequencies. The individual peak frequency was estimated as the median center frequency of the highest oscillatory peak detected within the broad band (alpha: 7–14 Hz, beta: 14–30 Hz) across all channels and resting state recordings. Similar to previous studies (Donoghue, Haller, et al. 2020; Hill et al. 2022), only alpha and beta oscillations were consistently detected in all participants (see Supporting Information S1).

### 2.5 HRV Data Processing

Due to distortions in ECG signals during simultaneous EEG and fMRI recordings, we opted for pulse‐to‐pulse intervals from PPG waveforms to estimate HRV parameters. PPG serves as a well‐established alternative to ECG for HRV evaluations, particularly under resting state conditions (Lu et al. 2009; Podaru et al. 2018; Schäfer and Vagedes 2013). Pulse waves were identified using a template‐matching approach (Elgendi 2012; Papini et al. 2018). In detail, raw PPG signals underwent band‐pass filtering between 0.5 and 10 Hz with a fourth‐order Chebyshev II filter (Liang et al. 2018), and respiratory‐induced amplitude variations were removed through envelope‐based demodulation (Charlton et al. 2016).

The resulting signals were segmented using local maxima of the second derivative as candidate pulse onset locations. Each candidate pulse waveform was compared with a template derived from the 10 surrounding pulses. Signal quality was assessed using the Root Mean Square Error (RMSE) (Awodeyi et al. 2014), and pulses with RMSE values surpassing a threshold of 0.2 were excluded. Subsequently, the signals were manually examined for quality assurance. The inter‐beat intervals (IBIs) were then obtained as peak‐to‐peak intervals from the resulting pulse waves. Erroneous IBIs, such as those from ectopic beats or false/missed beat detections, were corrected using an automatic artifact correction algorithm (Lipponen and Tarvainen 2019).

Following this, time‐, frequency‐, and geometric‐domain HRV parameters were computed. The time‐domain parameter root mean square of successive differences between adjacent IBI intervals (RMSSD) was utilized. In the geometric domain, Baevsky's stress index (SI) (Baevsky and Chernikova 2017; Tarvainen et al. 2014) was computed. For the frequency domain, the ratio between power in low frequency (LF, 0.04–0.15 Hz) and high frequency (HF, 0.15–0.40 Hz) LF/HF ratio was obtained (Malik et al. 1996). For this, the IBI time course was de‐trended using the smoothness priors‐based approach (Tarvainen et al. 2002), and spectral power was calculated using the Lomb‐Scargle periodogram. LF/HF ratios were log‐transformed before statistical analysis to meet normality assumptions.

### 2.6 Statistical Analysis

To assess the impact of psychosocial stress on the aperiodic and oscillatory activity of resting state EEG, we employed cluster‐based permutation testing (Maris and Oostenveld 2007), implemented in the FieldTrip toolbox (Oostenveld et al. 2011). This approach, controlling for multiple comparisons and devoid of prior assumptions about effect location, considers the adjacency of evaluated samples in spatial‐ and temporal‐spectral domains. Differences across all electrodes and frequency bins for oscillatory power spectra between pre‐stress RS1 and post‐stress RS2 under placebo and Nx4 were evaluated using two‐tailed paired *t*‐tests. Clusters were formed with ≥ 3 neighboring electrodes significant at *p* < 0.05, employing an electrode's neighborhood based on a triangulation approach (Oostenveld et al. 2011). Cluster values were determined by summing the t‐values of all samples within the cluster. The statistical significance of clusters was determined through 5000 Monte Carlo randomizations using a 0.05 significance level. In line with recent recommendations (Meyer et al. 2021), we present the maximum *t* statistic within each cluster, Cohen's *d* averaged across all electrodes within each cluster, and the *p*‐value for the cluster. To compare placebo and Nx4 for stress‐induced changes, ΔRS2‐RS1 was calculated separately for placebo and Nx4, followed by cluster‐based permutation tests.

For HRV analysis, a repeated measure analysis of variance (RM‐ANOVA) with two within‐subject factors of stress (RS1 pre‐stress/RS2 post‐stress) and treatment (Placebo/Nx4), and a between‐subject factor treatment sequence (Placebo‐Nx4/Nx4‐Placebo), was applied. If the assumption of sphericity, as indicated by Mauchly's Test, was violated, the Greenhouse–Geisser correction was employed. Post hoc Šídák's multiple comparisons tests were conducted to compare pre‐stress RS1 to post‐stress RS2 under placebo or Nx4. To compare placebo and Nx4 for stress‐induced changes, ΔRS2‐RS1 was calculated separately for placebo and Nx4, followed by paired *t*‐tests.

In the correlation analysis of the Nx4 effect on stress‐induced changes in EEG and HRV, the Nx4 effect measure on stress‐induced changes was defined as ((Placebo: RS2–RS1) ‐ (Nx4: RS2–RS1)). Channel‐wise Pearson linear correlations with cluster‐based permutation (Maris and Oostenveld 2007) were applied, forming clusters with a minimum of 2 channels and using a *p*‐value < 0.05 threshold. Significance was determined through 5000 Monte Carlo permutations.

## 3 Results

### 3.1 The Stress‐Induced Decrease in EEG Aperiodic Offset Was Significantly Mitigated by Nx4

We employed cluster permutation statistics to compare EEG parameters, including the offset (broadband shift) and exponent (slope) of aperiodic activity, oscillatory power at each frequency, and individual‐adjusted mean oscillatory alpha and beta power, between post‐stress and pre‐stress resting state recordings (RS2 vs. RS1) under both placebo and Nx4 conditions.

**FIGURE 1:**
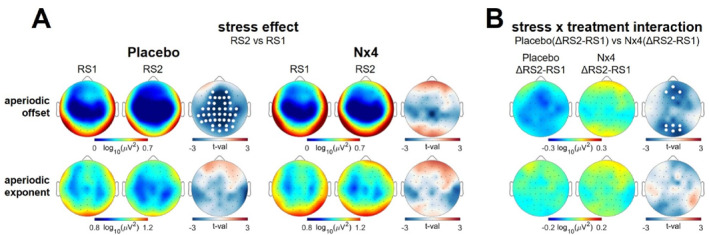
Aperiodic EEG activity in placebo and Nx4 conditions. (A) Scalp distribution of aperiodic offset (top row) and aperiodic exponent (bottom row) for pre‐stress resting state (RS1) and post‐stress resting state (RS2), along with t‐values resulting from the comparison of post‐stress RS2 and RS1 for placebo and Nx4 conditions. White dots indicate significant clusters from pairwise permutation tests (*p* < 0.05, 5000 permutations). The aperiodic offset decreased from pre‐stress RS1 to post‐stress RS2 in the placebo condition, while the decrease was less pronounced for Nx4. (B) Scalp distributions of mean stress‐induced (ΔRS2‐RS1) change in aperiodic offset (top row) and aperiodic exponent (bottom row) for placebo and Nx4, along with t‐values resulting from comparing placebo and Nx4 conditions. The stress‐induced decrease in aperiodic offset was significantly diminished in the Nx4 condition compared to the placebo.

Cluster‐based tests revealed a widespread decrease in aperiodic offset (tmax = −4.64, dmean = 0.31, *p* = 0.003) from pre‐stress (RS1) to post‐stress (RS2) resting states in the placebo condition. This reduction was observed across frontal, temporal, parietal, occipital, and central electrodes (Figure 1A upper left panel).

Conversely, in the Nx4 condition, aperiodic offset exhibited greater similarity between pre‐ and post‐stress resting states, with no significant clusters indicating decreased aperiodic offset (Figure 1A upper mid panel). To quantify the stress‐induced changes in aperiodic offset (ΔRS2‐RS1), we compared placebo and Nx4 conditions. The cluster‐based test identified two clusters with a significantly diminished stress effect of Nx4 compared to placebo (Figure 1B upper right panel): parieto‐occipital (tmax = −2.99, dmean = 0.04, *p* = 0.027) and frontal (tmax = −2.91, dmean = 0.03, *p* = 0.042). In essence, Nx4 mitigated the stress‐induced decrease in aperiodic offset.

The analysis did not uncover any noteworthy alterations in the aperiodic exponent from pre‐stress to post‐stress resting states. This parameter remained unaffected by both the stress task and Nx4, as depicted in the lower panel of Figure 1.

### 3.2 Stress‐Induced Elevation in Oscillatory Alpha Power in the Post‐Stress Resting State EEG Was Not Significantly Affected by Nx4

**FIGURE 2:**
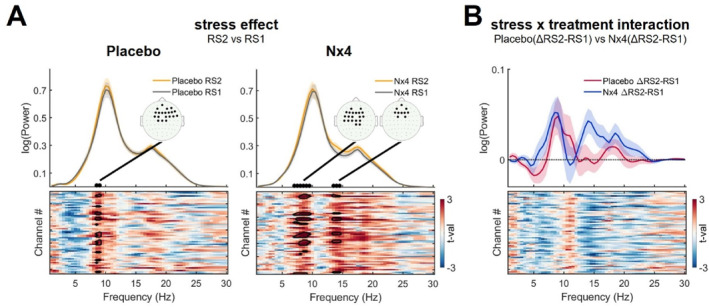
Oscillatory EEG activity in placebo and Nx4 conditions. (A) Top row: Oscillatory power during pre‐stress resting state RS1 (gray) and post‐stress resting state RS2 (orange) for placebo and Nx4 conditions. Solid lines indicate the mean power spectrum across all electrodes and participants, while shadows represent the standard error of the mean. Bottom row: Matrix depicting the t‐values resulting from comparing power at each frequency (*x*‐axis; 1–30 Hz) and electrode (*y*‐axis) between post‐stress RS2 and pre‐stress RS1. Solid contours indicate significant clusters from pairwise permutation tests (*p* < 0.05, 5000 permutations). Topographical plots depict electrode locations for significant clusters. Fronto‐central alpha power increased from pre‐stress RS1 to post‐stress RS2 in both placebo and Nx4 conditions. (B) Top row: Mean stress‐induced (ΔRS2‐RS1) oscillatory power change (solid line) and standard error of mean (shaded) for placebo (red) and Nx4 (blue) conditions. Bottom row: Matrix depicting the t‐values resulting from comparing stress‐induced (ΔRS2‐RS1) oscillatory power change at each frequency (*x*‐axis; 1–30 Hz) and electrode (*y*‐axis) between placebo and Nx4 conditions.

Cluster‐based tests revealed an increase in oscillatory power within the 8.5–9 Hz frequency range in the fronto‐central electrodes from pre‐to post‐stress resting states under the placebo condition (tmax = 3.68, dmean = 0.44, *p* = 0.039; Figure 2A left). In the Nx4 condition, two clusters indicated an increase in oscillatory power in post‐stress resting state RS2 within the frequency ranges of 7–9.5 Hz (tmax = 5.48, dmean = 0.49, *p* = 0.008) and 13.5–14.5 Hz (tmax = 3.67, dmean = 0.46, *p* = 0.044) in the fronto‐central location (Figure 2A right). However, the comparison of stress‐induced changes (ΔRS2‐RS1) between placebo and Nx4 conditions remained non‐significant (Figure 2B).

**FIGURE 3:**
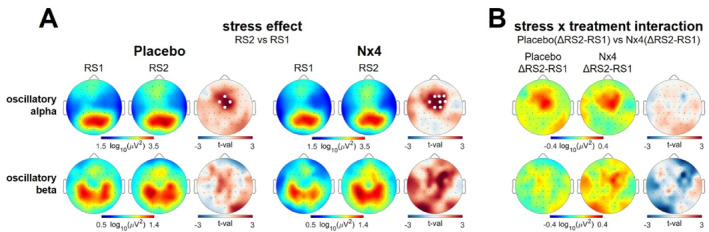
Mean oscillatory power in placebo and Nx4 conditions. (A) Scalp distribution of mean oscillatory alpha (top row) and beta (bottom row) power for pre‐stress resting state (RS1) and post‐stress resting state (RS2), along with t‐values resulting from the comparison of post‐stress RS2 and RS1 for placebo and Nx4 conditions. Fronto‐central alpha power increased from pre‐stress RS1 to post‐stress RS2 in both the placebo and Nx4 conditions. (B) Scalp distributions of mean stress‐induced (ΔRS2‐RS1) change in oscillatory alpha (top row) and beta (bottom row) for placebo and Nx4, along with t‐values resulting from comparing placebo and Nx4 conditions. White dots indicate significant clusters from pairwise permutation tests (*p* < 0.05, 5000 permutations). Individual‐adjusted mean oscillatory alpha and beta power were extracted using a ± 2 Hz interval around the individual peak frequency.

In an additional analysis of mean oscillatory power, estimated based on subject‐specific frequency ranges, an increase in oscillatory alpha power was observed in the fronto‐central electrodes (Figure 3 upper row). A significant stress effect was evident in both the placebo (tmax = 3.22, dmean = 0.03, *p* = 0.036) and the Nx4 condition (tmax = 4.02, dmean = 0.06, *p* = 0.014). No significant difference in stress induction was observed between placebo and Nx4. As for oscillatory beta power, no significant changes were observed between pre‐ and post‐stress resting states, nor between placebo and Nx conditions (Figure 3 lower row).

### 3.3 Nx4 Ameliorated the Stress‐Induced Increase in LF/HF Ratio and SI but Not the Stress‐Induced Reduction in RMSSD

Three distinct Heart Rate Variability (HRV) parameters, representing frequency, geometric, and time domains, were derived from pre‐ and post‐stress resting state PPG signals: LF/HF ratio, SI, and RMSSD, respectively. Significant main effects of stress were observed for all three HRV parameters ‐ LF/HF ratio (F(1,35) = 10.682, *p* = 0.002, ƞ2 = 0.234), SI (F(1,35) = 16.176, *p* < 0.001, ƞ2 = 0.316), and RMSSD (F(1,35) = 12.885, *p* = 0.001, ƞ2 = 0.269).

**FIGURE 4:**
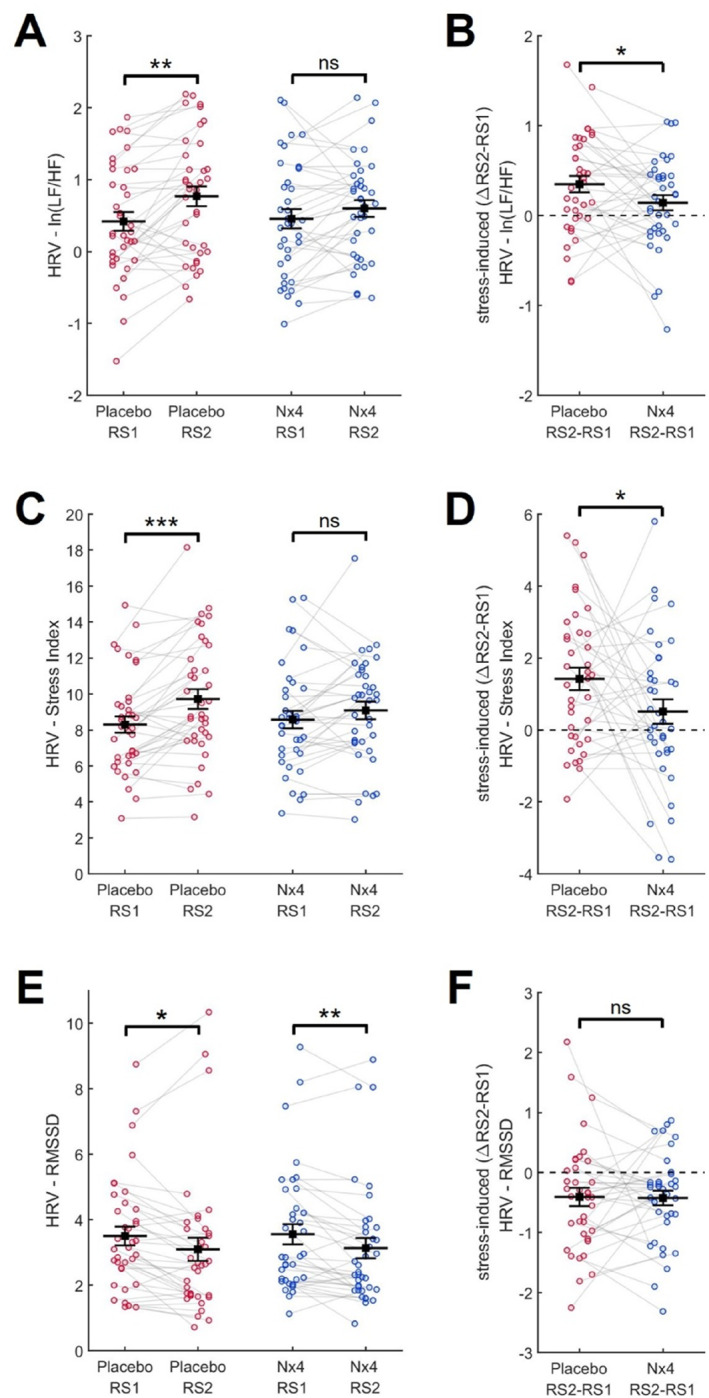
HRV Parameters LF/HF Ratio, Baevsky's Stress Index, and RMSSD in Placebo and Nx4 Conditions. (A) The LF/HF ratio increased from pre‐stress resting state 1 (RS1) to post‐stress resting state 2 (RS2) in the placebo condition, whereas it exhibited a lesser increase for Nx4. (B) The stress‐induced increase in LF/HF ratio was significantly diminished in the Nx4 condition compared to the placebo. (C) Baevsky's Stress Index increased from pre‐stress RS1 to post‐stress RS2 in the placebo condition, with a less pronounced increase observed for Nx4. (D) The stress‐induced increase of Baevsky's Stress Index was significantly diminished in the Nx4 condition compared to the placebo. (E) RMSSD decreased from pre‐stress RS1 to post‐stress RS2 in both the placebo and Nx4 conditions. (F) The stress‐induced decrease in RMSSD did not exhibit significant differences between the placebo and Nx4 conditions. Data are presented as individual dot plots with mean ± standard error of the mean. Statistically significant differences between RS1 and RS2 within the placebo or Nx4 condition (A, C, E), as well as between placebo and Nx4 for the stress‐induced (ΔRS2‐RS1) changes (B, D, F), are indicated by asterisks (*p < 0.05; **p < 0.01; ***p < 0.001; ns not significant).

In the placebo condition, the LF/HF ratio, indicative of the global sympatho‐vagal balance, increased from pre‐to post‐stress resting state (*p* < 0.001, *d* = 0.629; Figure 4A), reflecting a shift toward a sympathetic tone in the autonomic nervous system (ANS). Similarly, SI, a measure of sympathetic regulation, was significantly higher in post‐stress RS2 compared to pre‐stress RS (*p* < 0.001, *d* = 0.745; Figure 4C). Correspondingly, the parasympathetic RMSSD decreased significantly from pre‐to post‐stress resting states (*p* = 0.011, *d* = −0.437; Figure 4E).

Concerning the impact of Nx4 treatment versus placebo, we observed a statistically significant stress × treatment interaction for LF/HF (F(1,35) = 4.229, *p* = 0.047, ƞ2 = 0.109). The stress treatment x interaction for SI was just below the statistical significance threshold (F(1,35) = 3.790, *p* = 0.060, ƞ2 = 0.098).

In contrast to the placebo condition, both the LF/HF ratio and the SI did not exhibit significant differences between pre‐stress RS1 and post‐stress RS2 for the Nx4 condition (Figure 4A and 4C). When comparing stress‐induced changes in LF/HF ratio (Figure 4B) and SI (Figure 4D), defined as ΔRS2‐RS1, we observed significantly diminished stress effects in Nx4 compared to placebo (LF/HF: *p* = 0.0416, *d* = 0.347; SI: *p* = 0.0499, *d* = 0.334).

For RMSSD, no discernible difference between the placebo and Nx4 conditions emerged. In both cases, placebo and Nx4, RMSSD decreased from pre‐to post‐stress resting states (Figure 4E), and no distinction between placebo and Nx4 was observed for the stress‐induced change in RMSSD (Figure 4F).

### 3.4 Nx4 Effects on Stress‐Induced Change of Aperiodic Offset and SI Correlated

**FIGURE 5:**
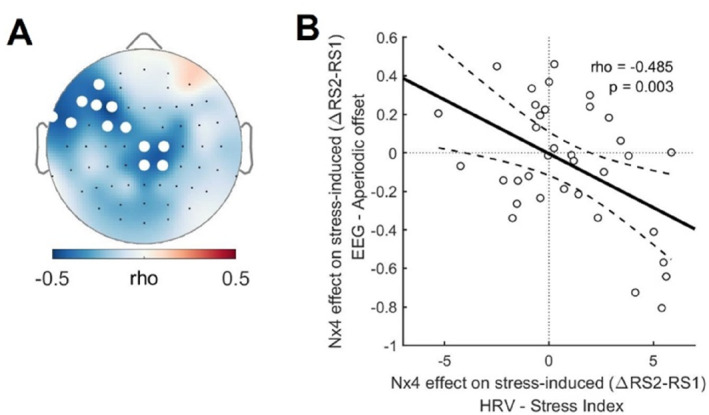
Correlation between Nx4 effects on stress‐induced changes in aperiodic offset and Baevsky's Stress Index. (A) Topographical map displaying the Pearson linear correlation coefficient between Nx4 effects on stress‐induced changes in aperiodic offset and stress‐induced changes in Baevsky's Stress Index. White dots indicate significant clusters from permutation tests (*p* < 0.05, 5000 permutations). (B) Correlation plot illustrating the mean Nx4 effect on stress‐induced changes in aperiodic offset, averaged across all significant electrodes within the cluster, and the stress‐induced change in Baevsky's Stress Index.

To assess the correlation between Nx4 effects on stress‐induced changes in EEG and HRV, we defined the Nx4 effect measure on stress‐induced changes as [(Placebo: RS2–RS1) ‐ (Nx4: RS2–RS1)]. Subsequently, we employed channel‐wise Pearson linear correlation with cluster‐based permutation testing to determine whether Nx4‐induced reductions in EEG and HRV stress reactivity were correlated. We identified a significant negative correlation between the Nx4 effect on stress‐induced changes in SI and the offset of aperiodic EEG activity (rhomax = −0.48, *p* = 0.012) (Figure 5). This negative correlation indicates that the stress‐reducing effect of Nx4 is observed concurrently in both EEG, as reflected by the aperiodic offset, and HRV, as reflected by SI.

## 4 Discussion

In this post hoc analysis of data from an exploratory clinical trial, we investigated the impact of acute psychosocial stress on post‐stress resting state HRV and EEG signals. We also explored whether the anti‐stress medication Nx4 could mitigate these stress‐induced changes in both peripheral physiological HRV and central electrophysiological EEG readouts.

Due to the requirements of the primary endpoints of the trial based on fMRI (Herrmann et al. 2020), several technical challenges for HRV and EEG readouts had to be addressed: For the psychosocial stress induction, a modified version of the TSST adapted for use inside an MRI scanner, the ScanSTRESS was applied as described previously (Herrmann et al. 2020; Streit et al. 2014). Interferences from the MRI on the EEG signals have been overcome by using CWLs that increased the quality of the EEG recordings inside the scanner. Unfortunately, the ECG signals that were collected in parallel to the EEG/fMRI recordings were heavily distorted and unusable for assessing HRV. Therefore, we used PPG data that were acquired using an MRI‐compatible plethysmograph as an alternative to ECG for HRV evaluations (Lu et al. 2009; Podaru et al. 2018; Schäfer and Vagedes 2013).

We found that the stress‐task‐induced physiologic adaptations in the periphery as well as in the brain were readily detectable in the resting‐state recovery phase after the stress paradigm: The aperiodic component of EEG signals, which represents ongoing, non‐oscillatory neural activity, was reduced. This reduction might be interpreted as a sign of heightened arousal due to psychosocial stress that persisted from the stress task into the immediately following resting state. Recent research indicates that increased arousal or stress is associated with reductions in aperiodic EEG components, particularly a flattening of the aperiodic slope. For instance, Lendner et al. (2020) demonstrated such associations across sleep‐wake transitions. More recently, Brandes‐Aitken et al. (2023) found that higher maternal hair cortisol levels, indicative of chronic stress, were associated with alterations in infants' frontal EEG activity, including a flattening of the aperiodic spectral slope, suggesting that maternal stress may influence the development of infants' neural arousal regulation mechanisms. In parallel, the oscillatory alpha power was increased which can be interpreted as a shift toward internal processing such as mind wandering (Compton et al. 2019). On the peripheral level, we observed that the global sympato‐vagal balance was shifted toward the SNS as shown by increased HRV readouts LF/HF ratio and SI, as well as a decreased RMSSD reflecting the parasympathetic branch of the ANS. The intake of a single dose of Nx4 reduced these stress‐induced changes both on the peripheral physiological (HRV) and central electrophysiological (EEG) levels. Nx4 effects on aperiodic offset and SI were significantly correlated. Although subjective nervousness ratings were not analyzed in the current manuscript, prior results from the same trial (Herrmann et al. 2020) using a visual analogue scale (VAS) confirmed that participants experienced increased stress after the ScanSTRESS task. However, no effect of Nx4 on VAS ratings was observed. This absence of correspondence with physiological markers is consistent with previous research showing that subjective and objective stress indicators often diverge, as they reflect different underlying processes—conscious appraisal versus automatic regulation (Campbell and Ehlert 2012; N. Ali et al. 2017).

### 4.1 Stress Led to Increased LF/HF and SI Significantly Reduced by Nx4

At the peripheral physiological level, we observed a significant rise in the LF/HF ratio and the SI as well as a reduction of the RMSSD following exposure to psychosocial stress. The LF/HF ratio is an established measure of the global sympato‐vagal balance (Shaffer and Ginsberg 2017). HF power is indicative of PNS activity responsible for maintaining internal functions during rest (Squire 2009). Decreased HF power is commonly associated with stress, panic, anxiety, or worry (Shaffer and Ginsberg 2017). LF power, representing a mix of sympathetic and parasympathetic influences, predominantly signifies sympathetic activity (Malliani et al. 1991; Ori et al. 1992). The SNS, vital for maintaining homeostasis during stress exposure (Squire 2009), contributes to the stabilization of heart rhythm (M. K. Ali et al. 2021). The SI, an index characterizing the activity of sympathetic or central regulation, is particularly sensitive to changes in sympathetic tone (Baevsky and Chernikova 2017). As per these definitions, an anticipated increase in both LF/HF ratio and SI after stress is expected, serving as markers of SNS activation. Conversely, RMSSD, related to the parasympathetic branch of the ANS, tends to be downregulated in stressful situations. Accordingly, a decreased RMSSD was observed in the post‐stress resting state of this study.

Interestingly, the anti‐stress medication Nx4 mitigated the stress effects on LF/HF and SI but did not exhibit the same influence on RMSSD. This observation suggests that the impact of Nx4 may be more closely associated with the sympathetic rather than the parasympathetic branch of the ANS. The attenuating effect of Nx4 on the SNS harmoniously aligns with its documented influence on the amygdala. The amygdala is primarily linked to the activation of the SNS and the initiation of the “fight or flight” response during stress. It plays a pivotal role in detecting and processing emotionally salient stimuli, including potential threats, orchestrating a cascade of responses aimed at preparing the body for action. In the NEURIM trial, Nx4 demonstrated a reduction in amygdala activation in response to negative emotional stimuli (Herrmann et al. 2020) and diminished the stress‐induced elevation of functional connectivity between the amygdala and anterior cingulate cortex, which are components of the brain stress network (Nanni‐Zepeda et al. 2022).

### 4.2 Stress Led to a Decreased Aperiodic Offset Which Was Ameliorated by Nx4

At the neural level, we observed a reduction in the offset of the aperiodic (1/f‐like) EEG component, along with an increase of fronto‐central oscillatory alpha power during the resting state following exposure to stress.

The impact of stress on oscillatory alpha power is subject to varied discussions. Alpha power is often reported to decrease with stress (Vanhollebeke et al. 2022), especially during acute stress induction (Giannakakis et al. 2022). However, others have found no difference in alpha power between pre‐ and post‐stress resting states (Berretz et al. 2022). Intriguingly, increased alpha power has been demonstrated during the resting state following cognitively demanding tasks (Magosso et al. 2019; Mathewson et al. 2015; Simon et al. 2011). This elevation in alpha power may signify ongoing engagement in internal cognitive processing, such as the mental arithmetic involved in the ScanSTRESS task. Alternatively, it could be interpreted as a shift toward mind wandering (Compton et al. 2019). The acute psychosocial stress in ScanSTRESS may suppress the availability of cognitive resources, fostering an internally oriented focus of attention, particularly in participants with a negative mood (Vinski and Watter 2013). Notably, unintentional mind wandering has been correlated with symptoms of depression, anxiety, and stress (Seli et al. 2019). Consequently, the observed increase in fronto‐central oscillatory alpha power may be linked to exhaustion after ScanSTRESS. However, although the increased alpha oscillation can be explained as an expected stress reaction based on previous literature, we did not observe a significant effect of Nx4 on this particular neural readout.

We did, however, observe an impact of Nx4 on the stress‐induced reduction of aperiodic offset. While classical EEG studies have predominantly relied on Fast Fourier Transform (FFT) ‐ based band power estimates, Frequency Offset and Order Fitting (FOOF) offers specifically identify and characterize oscillations (peaks) within the spectrum, accounting for the aperiodic component. The FOOOF algorithm allows for this decomposition and addresses a key limitation of traditional methods by isolating oscillatory peaks from the aperiodic background. The aperiodic spectral offset is theorized to closely monitor the activation level at cortical circuits (Manning et al. 2009; Miller et al. 2014). It has been demonstrated to positively correlate with the activity of the posterior salience network and negatively with the activity of the frontal cortex (Jacob et al. 2021), regions associated with the stress response. Reduced aperiodic offset, coupled with increased cortisol levels, has been noted during long‐term isolation (Weber et al. 2020), both of which returned to baseline levels afterward. This implies a potential link between broadband EEG power and stress‐related processes. A recent study reported a progressive increase in aperiodic offset from wakefulness to deep sleep (Favaro et al. 2023). Aperiodic offset also increased after sleep deprivation, which has been associated with decreased subjective alertness, highlighting the offset's sensitivity to changes in arousal and vigilance states (Bai et al. 2024). Consequently, the decreased aperiodic offset during the post‐stress resting state in our analysis might reflect elevated arousal levels. Indeed, a hyperalert state of the NEURIM study participants in the post‐stress resting state has been previously reported (Chand et al. 2021). The hyperarousal was significantly diminished by Nx4 treatment, aligning with the reduced decrease in aperiodic offset in our analysis.

This finding also aligns well with the Nx4 effect on stress‐induced LF/HF and SI, as arousal and the SNS are closely related. Activation of the SNS induces various physiological responses aimed at preparing the body for action and contributes to an aroused state. The interconnectedness of these systems concerning stress and the Nx4 effect is further reflected in the observation that the Nx4 effects on stress‐induced reduction of aperiodic offset significantly correlated with the effect on stress‐induced increase of SI. This correlation emphasizes the relationship between the central and peripheral effects of Nx4.

### 4.3 Limitations

All results reported in this article are exploratory and do not aim to make any confirmatory claims. Due to the post hoc nature of the analysis presented here, these specific analyses were not pre‐registered and no a priori sample size calculation was performed specifically for these analyses. As this was an exploratory analysis without pre‐registration, the risk of false positive findings cannot be excluded, especially given the number of endpoints examined. The results should therefore be interpreted as hypothesis‐generating, and replication in a pre‐registered, adequately powered study is warranted.

All participants were male and were selected based on stress scores to ensure susceptibility to stress without chronic stress, thus avoiding a ceiling effect in sensitivity to experimental stress induction. The exclusion of female participants and participants with extreme stress scores in either direction limits the generalizability of our results.

Although physiological data were recorded during the ScanSTRESS itself, these data were not included in the present analyses. The aim of these analyses was to characterize resting EEG and HRV activity before and after stress induction, aligning with our primary goal of assessing neurophysiological markers of recovery and treatment effects. However, analyzing data acquired during the stress task could provide valuable complementary insights into dynamic physiological responses to acute stress and should be addressed in future work.

## 5 Conclusion

In this post hoc analysis of clinical trial data, we found that stress induced by a modified version of the TSST resulted in a decreased EEG aperiodic offset, which we speculate represents heightened arousal and an increased alpha oscillation, suggesting a shift toward internal processing. Additionally, there were elevated HRV parameters LF/HF and SI, reflecting sympathetic activation along with a decreased RMSSD, indicative of parasympathetic activity. Nx4 demonstrated a comprehensive impact on both peripheral physiological and central electrophysiological levels, mitigating the stress‐induced effects by restoring pre‐stress levels of LF/HF, SI, and EEG aperiodic offset. The noteworthy correlation between Nx4 effects on aperiodic offset and SI suggests a potential shared underlying mechanism contributing to these improvements in the stress response, aligning with previously reported effects of Nx4.

## Author Contributions

**Marina Krylova:** writing – original draft, formal analysis, software, methodology. **Sarah Alizadeh:** formal analysis, writing – review and editing. **Hamidreza Jamalabadi:** formal analysis. **Igor Izyurov:** formal analysis. **Tara Chand:** formal analysis, writing – review and editing. **Johan van der Meer:** investigation. **Johannes C. Vester:** formal analysis, conceptualization. **Britta Naschold:** writing – review and editing. **Myron Schultz:** conceptualization, writing – review and editing. **Veronika Engert:** writing – review and editing. **Martin Walter:** conceptualization, methodology, supervision, writing – review and editing.

## Conflicts of Interest

Martin Walter received institutional research support from Heel paid to his institution for this study, and from Brain‐Wave Bank, HMNC, Perception Neuroscience and H. Lundbeck A/S outside the submitted work. The University of Tübingen, via MW, received institutional fees for advisory services by M.W. from Heel GmbH, Servier Deutschland GmbH, Bayer AG, and Janssen. MW further received compensation by the companies Janssen, Heel and Takeda for consulting and speaker activities.

Marina Krylova, Sarah Alizadeh, Hamidreza Jamalabadi, Igor Izyurov, Tara Chand, Johan van der Meer, and Veronika Engert, were part of Martin Walters's team for this study, and declare no other known competing financial interests or personal relationships that could have appeared to influence the work reported in this paper.

Johannes C. Vester is a senior biometric consultant of idv Datenanalyse & Versuchsplanung who was contracted by Heel for conceptualization and formal analysis of the NEURIM trial. Johannes C. Vester received personal fees for biometric services from the Foundation of the Society for the Study of Neuroprotection and Neuroplasticity (SSNN) outside the submitted work, and idv Datenanalyse & Versuchsplanung received payments for biometric services from Heel, University Medical Center Göttigen, IgNova GmbH, Abnoba GmbH, AOP Orphan Pharmaceuticals AG, IDEA AG, PBB Entrepreneur Ltd, Tillots Pharma AG, STORZ Medical AG, EVER Neuro Pharma GmbH, MUCOS Pharma GmbH & Co. KG, Steigerwald Arzneimittelwerk GmbH outside the submitted work.

Myron Schultz and Britta Naschold are employees of Heel GmbH.

## Supporting information


Supporting Information S1

## Data Availability

Data sharing not applicable to this article as no datasets were generated or analyzed during the current study.
