# Supplementary material for: Comprehensive Mitigation of Peripheral and Central Stress Responses by Nx4: Insights From EEG and Heart Rate Variability in Post‐Stress Resting State
Source: Hum Psychopharmacol. 2025 Oct 7;40(6):e70020. doi: 10.1002/hup.70020 (PMC12504797; doi:10.1002/hup.70020)
Supplement: Supplementary file 1 — Supporting Information S1 [file HUP-40-e70020-s001.pdf]

Comprehensive mitigation of peripheral and central stress responses by Nx4: Insights from EEG and heart rate variability in post-stress resting state

Supplementary data

Supplementary figures S1 to S5.

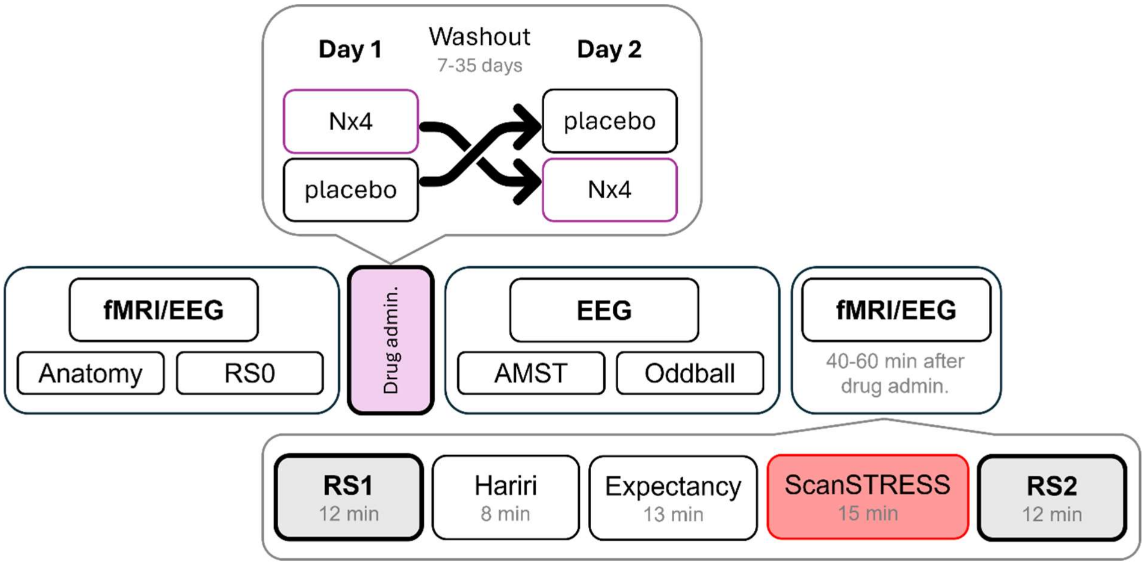

Supplementary Figure S1: Experimental timeline of the cross-over clinical trial. Each study day began 20 minutes before drug administration with a combined fMRI/EEG recording, including a 5-minute anatomical scan and a 12-minute resting-state measurement (RS0). At time point 0, participants received either Nx4 or placebo in a randomized cross-over design, with a washout period of 7–35 days between Day 1 and Day 2. Following drug administration, an EEG session was conducted, comprising the AMST (Attention Modulation by Salience Task) and the Oddball paradigm. Between 40- and 60-minutes post-administration, participants underwent a second fMRI/EEG session, which included a 12-minute resting-state scan (RS1) before the task-based measurements (Hariri task: 8 min; Expectancy task: 13 min; ScanSTRESS paradigm: 15 min), and a second 12-minute resting-state scan (RS2) after completion of the stress inducing tasks.

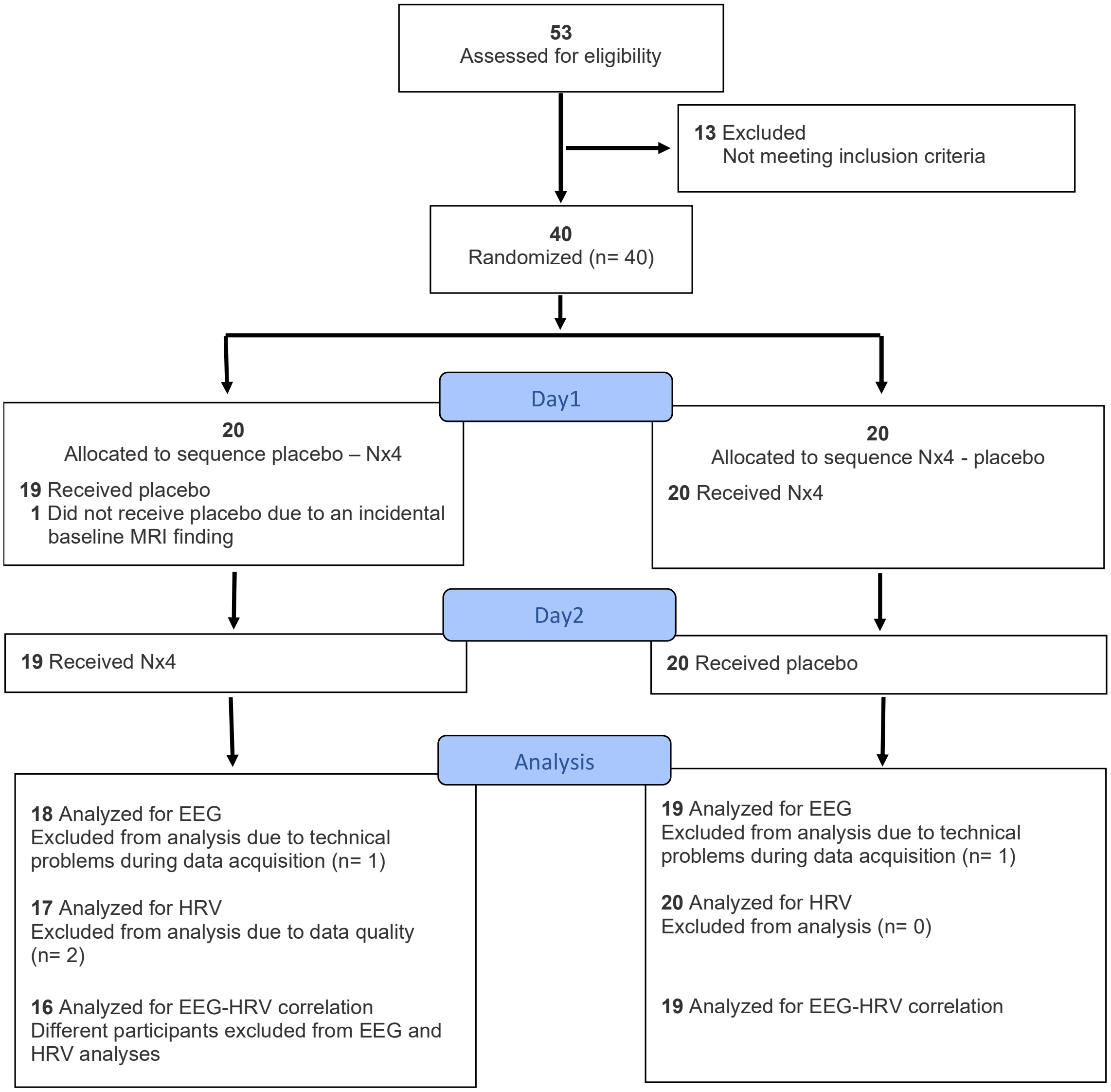

Supplementary Figure S2: Flow diagram showing number of participants through each stage of the randomized crossover trial.

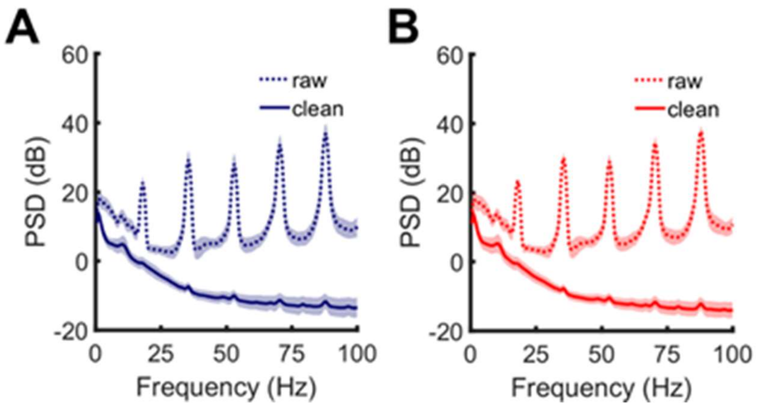

Supplementary Figure S3: EEG artifact correction quality. Mean power spectral density (PSD) across all subjects, channels, and all resting state recordings before (solid lines) and after (dashed lines) correction for helium pump and ballisto-cardiac artifacts in placebo (Panel A) and Nx4 (Panel B). Shadows represent the standard deviation across all subjects. We observe small residual artifacts in frequencies above 34 Hz and restrict the analysis to frequencies lower than 30Hz.

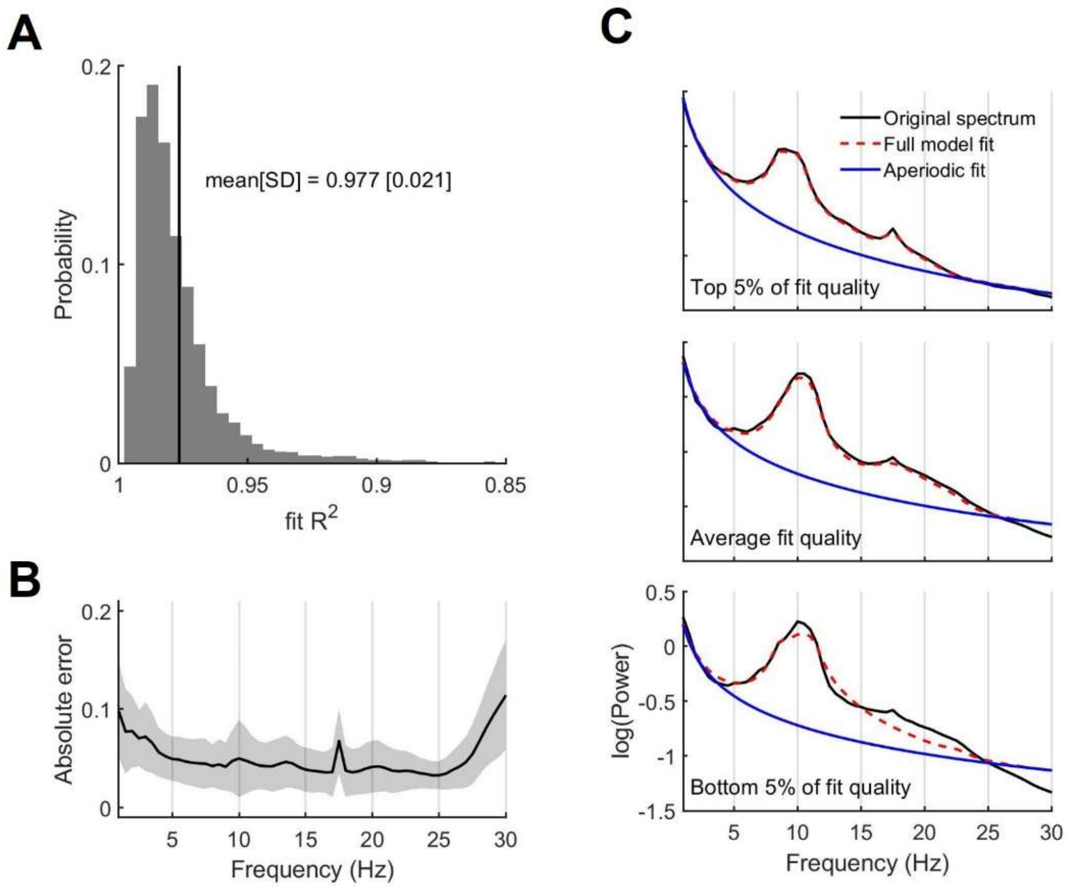

Supplementary Figure S4: EEG spectral parametrization - goodness of fit. Results are pooled across subjects, channels, resting state recordings, and measurement days. (**A)** Histogram of R^2 values between experimental and model spectra. Vertical lines indicate a mean R^2 of 0.977, associated with objectively good fits. (**B**) Group frequency-by-frequency absolute error of fit. The plot line indicates the mean and shadows represent the standard deviation across the cohort. Errors are relatively even across frequencies with a slight increase for frequencies above 25 Hz and a clear peak at 17.5 Hz, corresponding to residual gradient artifacts. **(C)** Examples of model fit quality. Plots are ordered from top to bottom based on decreasing goodness of fit and represent the original power spectrum (black), aperiodic (blue), and model fits averaged across 5% of fits with highest, lowest, and centered around the mean R^2.

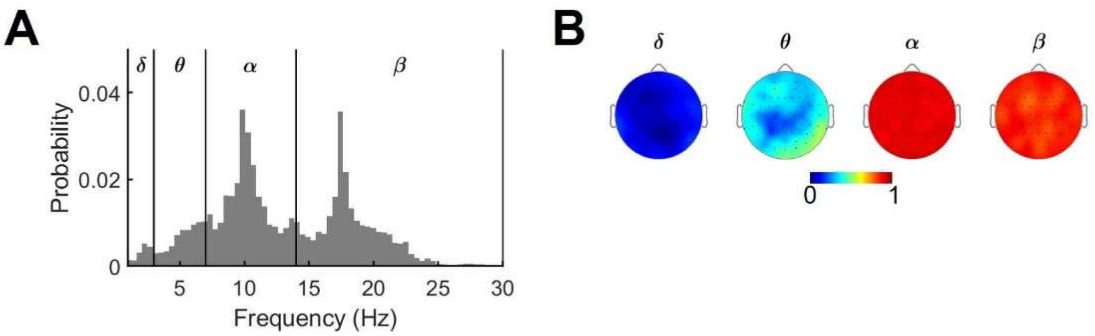

Supplementary Figure S5: Occurrence of periodic oscillation. **(A)** Distribution of center frequencies for all oscillatory peaks pooled across subjects, channels, resting state recordings, and measurement days. Two prominent peaks in the probability distribution correspond to canonical alpha and beta frequency bands. **(B)** Oscillation probability maps for four predefined frequency bands: delta (1-3 Hz), theta (3-7 Hz), alpha (7-14 Hz) and beta (14-30 Hz). Colors indicate the proportion of recordings with detected oscillation peaks within the specified frequency range at each electrode. Alpha and beta exhibit maximal values approaching 1, reflecting a high level of consistency across participants.
